# Cold flow properties of biodiesel from waste cooking oil and a new improvement method

**DOI:** 10.1016/j.heliyon.2024.e36756

**Published:** 2024-08-24

**Authors:** Abderrahim Bouaid, Gabriel Iliuta, Jorge Mario Marchetti

**Affiliations:** a Chemical Engineering Department, Faculty of Chemistry, University of Complutense, 28040, Madrid, Spain; b Faculty of Science and Technology, Realtek, Norwegian University of Life Sciences, Drøbakveien 31, 1432, Ås, Norway

**Keywords:** Biodiesel, Cold flow properties, Eversa 2.0, WCO, Optimization

## Abstract

Biodiesel despite its positive advantages when using it as a fuel in replacement of diesel, suffers a major drawback in the cold flow properties (CFP). During winters, plugging of the filters as well as crystallization of the fatty acid are two of the leading problems that makes the fuel to not reach injectors and the combustion chamber and therefore the engine does not start. Cold flow properties of waste cooking oil biodiesel (WCOB) through the reduction of cloud point (CP), pour point (PP) and the cold filter plugging point (CFPP) where investigated in this work. The effectiveness of an approaches using the combination of two techniques, controlled winterization and addition of fatty acids 2-ethylhexyl esters (FAEhE) to reduce CP, PP and CFPP was studied. The change in CP, PP and CFPP corresponded to a decrease in the saturated ethyl esters content. A reduction of the palmitic and stearic acid ethyl esters content of 20,63 % and 8.64 % respectively was found. There was not significant effect on the fuel properties due to changes in the chemical composition of liquid fractions. However, using a Factorial Design and Response Surface Methodology optimization, the lowest CP, PP and CFPP for WCOB biodiesel could be obtained working with a winterization temperature of −5 °C, adding a 10 % of FAEhE and cooling during 30 min.

## 1 Introduction

The feedstocks for biodiesel production vary significantly by location, influenced by climate and local availability. In the United States, soybean oil serves as the predominant source for biodiesel, while in Europe and tropical countries, rapeseed oil and palm oil are the primary feedstocks, respectively. Despite this, there are no technical barriers to utilizing other varieties of vegetable oils for biodiesel production [1].

The use of food-based biofuels has been debated for a long time, not only in relationships with the negative environmental and social impacts, but also in relation to the climate impact that is related to the use of land and the emissions produced. This is directly related to the need of agricultural land to be moved due to the use of locations for biofuel production. And due to the worlds growing demand for food and animal feed, this land pressure causes a substantial increase in CO_2_ emissions [2].

The push by the European Union for biodiesel derived from crops resulted in the emergence of a new market for agricultural commodities. During the period from 2005 to 2015, there was a decline in the consumption of vegetable oil within the food industry, dropping from 15.1 million tons to 13.7 million tons. Conversely, its use in the bioenergy sector saw a significant increase, nearly quadrupling from 2.9 million tons to 10.5 million tons [3].

According to Oil World data, 60 % of the rapeseed consumed in the European Union is utilized for biodiesel production, with the total volume remaining constant since 2009. During this period, only palm oil, the least expensive vegetable oil, has seen growth. In total, 44 % of all vegetable oils, including palm, soy, rapeseed, and sunflower oils, in Europe are used as feedstock for biodiesel production and are consumed in the process. Given the escalating issues, the European Union implemented a cap of 7 % on the inclusion of food-based biofuels towards the 10 % renewable energy target for transportation in 2015. This action aimed to mitigate the effects of Indirect Land Use Change (ILUC) [4]. It is probable that the existing political climate in Europe will further exacerbate this situation.

At present, the majority of production facilities rely on edible oils for manufacturing. In North America and Europe, canola, soybean, and rapeseed oils are the predominant oils used as raw materials, accounting for 70–95 % of the overall cost of producing biodiesel [5]. The increasing need for abundant supply of edible vegetable oils however has raised food security concerns and ethical “food vs fuel” issues [6].

In addition, the economic perspective for the biodiesel industry is not promising. It has a cost around 40 % more expensive that the equivalent from petroleum-based fuels. With a high cost related to the feedstocks being used and its high demand purity for conventional process-based technologies [7]. Consequently, finding and utilizing cost-effective and underexploited feedstocks for biodiesel production becomes crucial. Focusing on affordable production methods using non-edible feedstocks, such as non-edible vegetable oils [8], used cooking oils [9], and waste animal fats [10], referred to as second-generation biodiesel feedstocks—is now a significant area of research. This approach aims to lower fuel costs, manage highly contaminated waste oils, and advance the production of renewable energy while addressing climate change issues simultaneously. Furthermore, this will allow the use of waste sources where they are produced and no need for further transport or handling [11,12].

In this context, waste cooking oil (WCO), is a promising alternative for producing biodiesel because is a cheaper raw materials in comparison with refine oil. Furthermore, it does not use land for producing crops neither competes with edible oils that can be used for food purposes. Finally, the oil can be use directly for biofuel productions instead of being treated for disposal [13,14].

Recently, the use of enzymatic catalysis for biodiesel production, particularly with low-cost oils high in free fatty acids, has gained significant interest. This approach is favored primarily for its economic advantages, renewability, and sustainability benefits [15].

Utilizing enzymes as catalysts in the transesterification process offers numerous benefits compared to traditional catalysts. These include high selectivity, the ability to operate under mild conditions, minimal side reactions, and resulting in products of high purity [16].

Eversa Trasnform was launched by Novozymes as a commercially available liquid enzyme preparation from *Thermomyces lanuginosus* with enhanced ability to convert oils to biodiesel within 2015 [17]. These studies demonstrated enhanced enzyme recyclability, achieving up to 12 cycles of reuse, and higher biodiesel yields from soybean oil (over 97 %) and rapeseed oil (92–97 %) [17,18]. Utilizing the liquid enzyme Eversa Transform as a catalyst, without reusing the enzyme, led to a production cost of $0.78 per kg. This resulted in an annual profit of $51.6 million from biodiesel production totaling 250,000 tons [19]. An alternative alcohol to be used, instead of methanol, is ethanol, which will produce ethyl esters is of high interest due to the renewability and sustainability of this biodiesel [20].

Cold flow properties are of high relevance, special in the context of biodiesel, due to the high problems that could be brought when these properties are of low quality. This could lead to solidification of pure biodiesel in the fuel lines or clog filters when utilized in cold ambient conditions [21,22]. The poor performance of biodiesel at low temperatures is its major quality deficiency and the limiting factor to promote the use of this alternative fuel for pure diesel engines or blended in high proportions with conventional diesel fuel derived from petroleum [21,22].

Cloud point (CP), pour point (PP) and cold filter plugging point (CFPP) are the parameters to be determined to evaluate the properties on the cold flow properties. When biodiesel is subjected to lower temperatures there is the formation of solid wax crystal nuclei. Further decrease in temperature causes the crystal nuclei to grow [23] and become visible, and this temperature is termed as CP.

Very few works have been done in this area of interest, to improve the cold flow properties of biodiesel [[24], [25], [26]]. The cold flow properties of biodiesel can be improved in a variety of ways. Among the strategies identified to improve the low-temperature performance of biodiesel are the following.

### 1.1 Winterization

Winterization is a method now used to lower cloud points (CP) by decreasing the content of saturated alkyl esters. This process separates lipid components, such as vegetable oils, fats, fatty acids, fatty acid esters, mono-, di-glycerides, and their derivatives, according to their temperatures of crystallization [27].

Kumar and Sharma [28] presented a new process where a 3-step winterization process was used and they reported a reduction of CP by 5.5 °C and a reduction of PP by 8.2 °C. Even though the cold flow properties where reduced, there was also a reduction up to 15 % on the biodiesel yield due to this winterization approach. Which is counterproductive for the biodiesel quality due to a lower yield will also mean higher amount of impurities to be separated later.

### 1.2 Using additives

Leggieri et al. [29] applied dimethyl azelate and triacetin as additives to enhance the cold flow properties of fatty acid methyl esters (FAME), achieving a modest improvement in cloud point (CP) temperatures by 2–3 °C. Similarly, Monirul et al. [30] utilized poly(methyl acrylate) (PMA) to enhance the cold flow properties of biodiesel-diesel mixtures, observing improvements in pour point (PP), cloud point (CP), and cold filter plugging point (CFPP) by 9, 3, and 8 °C, respectively. A significant issue with these cold flow improvers (CFI) is their high cost, which in turn raises the overall cost of the fuel.

### 1.3 Blending with conventional diesel fuel

A third technique that can be used is blending. In this case, biodiesel can be blend with other petrochemicals components to improve the properties of the fuel. Kim et al. [31] explored the effects of cold temperatures (−16 °C and −20 °C) on different blends (B5, B10, and B20) of biodiesel fuels made from soybean oil, cottonseed oil, palm oil, and Jatropha oil in passenger car and light-duty truck engines. They discovered that the B5 blends were able to meet all test criteria under various conditions. However, the other blends encountered issues under certain testing scenarios.

Research in this field is essential, and further efforts are needed to enhance the cold flow properties of biodiesel. Within this study Eversa was used to produce biodiesel from waste cooking oil with high free fatty acid content. This process was selected to evaluate the performance of a commercial low-cost lipase. Ethanol was used in order to produce fatty acid ethyl esters.

Once the biodiesel was produced, cold properties of the biodiesel subjected to both methodologies (winterization and blending) were tested and evaluated. For the blending testing, lab-made alkyl esters were produced using Eversa and operational conditions previously tested. Therefore, this study investigates the enzymatic ethanolysis of WCO, determining the optimal conditions for various influencing factors through Factorial Design and Response Surface Methodology. This approach provides deeper insights per experiment compared to ad-hoc methods, allowing for the observation of interactions between experimental variables. This enhances the understanding of the process and helps reduce research time and costs. The manuscript novelty relies on the DOE and RSM of the cold fuel properties that to our knowledge has not yet been done and they are a crucial element when determining the usability of a fuel especially in cold weathers.

## 2 Material and methods

### 2.1 Equipment

A stirred batch reactor of 250 cm^3^ was used to carry out the all the transesterification reaction experiment. This reactor featured controls for temperature and stirring speed and was submerged in a water bath regulated by a thermostat. The bath's temperature was precisely monitored using a PID controller with an accuracy of 1 °C. The speed of the mechanical stirrer, powered by an IKA-labortechnik motor, was adjustable between 300 and 600 rpm, with an optimal speed of 350 rpm identified to effectively mitigate external mass transfer limitations [17]. Winterization studies were performed in a controlled crystallization bath, low temperature incubator (Heto lab. Equipment, Denmark) able to control working temperature down to −35 °C.

### 2.2 Materials

Waste cooking oil was kindly obtained from various restaurants in Madrid city. The fatty acid content of the oil was determined according to the AOCS official method. The result from this analysis and testing can be summarized in Table 1. Ethanol of 99.8 % purity was supplied by Panreac (Spain). The catalyst used, for transesterification reaction to transformed the waste cooking oil as well as to produce the FAEhE to be used as additive, was Novozyme Eversa, Transform 2.0 supplied by Novo Nordisk (Spain/Denmark). The fatty acids 2-ethylhexyl esters (FAEhE) used in the cold flow improvement studies were made in our lab by enzymatic transesterification of jojoba oil using branched (2-ethylhexanol) chain as alcohol, to produce jojobyl alcohol mixtures as high value-added products and obtaining as co-product, the FAEhE during the process, according to the process described in our published work [32].

**Table 1:** Characteristics of waste cooking oil used in this study and fatty acids composition.

Characteristics		Waste Cooking Oil
Acid number (mg kOH/g)		5.2
Iodine number (I_2_/100g)		118
Peroxide number (meq Per/kg)		7
Viscosity (40 °C)(mm^2^/s)		57
Water content (ppm)		240
Fatty acid compositions (%)		
Capric (C10:0)		–
Myristic (C14:0)		–
Palmitic (C16:0)		2.95
Stearic (C18:0)		8.89
Oleic (C18:1)		19.1
Linoleic (C18:2)		62.5
Linolenic (C18:3)		3.57
Eicosanoic (C20:0)		–
Henicosanoic (C20:1)		–
Other minor components		Rest to 100
***∑ saturated***		11.84
***∑ unsaturated***	mono	19.10
	poly	66.10

### 2.3 Procedure

#### 2.3.1 Fatty acids ethyl esters preparation

The process of transforming WCO into biodiesel was carried out through a transesterification reaction. The WCO was introduced into a reactor equipped with a reflux condenser. Upon reaching the target temperature, the catalyst and ethanol were heated separately and added. Subsequently, the speed of the impeller was set to its operational value, marking the commencement of the reaction time. Samples were periodically taken for analysis via gas chromatography. Throughout these experiments, the speed of the impeller and pressure were maintained consistently to prevent mass transfer limitations and process variability. A comprehensive description of the methodology can be found in the author's prior publications [17,33].

#### 2.3.2 Reaction time

Reaction time is a critical factor in any process, significantly impacting economic efficiency and energy use [34]. Initial experiments were conducted to determine the optimal reaction time. The kinetics of the transesterification reaction to produce biodiesel were observed over 7 h, with preliminary findings showing that equilibrium was achieved at 4 h, resulting in a 95.08 % conversion rate. No improvements in the yield of ethyl esters were observed with further increases in reaction time, leading to the conclusion that 5 h was the optimal duration for maximizing ethyl ester production. Therefore, a time of 5 h was set. Extending the reaction time beyond this point did not enhance the yield, possibly due to the reversal of the reaction that reduces the formation of fatty acid ethyl esters [35].

#### *2.3.3* Winterization and blending process

For this methodology, the biodiesel was agitated at 100 rpm during the winterization, this agitation was used to improve the mass transfer and the phase-based thermodynamic equilibrium. The temperature was reduced in intervals of 0.5 °C with a cooling rate of 0.5 C/min. The resulting crystals were then separated by vacuumed filtration and the samples produced from each filtration were analyzed in terms of cold flow properties (CP and PP), and saturated/unsaturated fatty acid composition. Furthermore, the crystallized biodiesel was weight to determine the yield of crystals produced. Phases where separated after the cooling time and the samples where analysis separately in a GC equipment.

Once the winterization has been carried out, blending of the outcome with FAEhE biodiesel produced from jojoba oil was performed. The blending was studied in range from 5 to 5 % in weight.

### 2.4 Analytical methods

The composition of fatty acid ethyl esters was analyzed using capillary column gas chromatography, specifically with a Hewlett–Packard 5890 series II system equipped with a flame ionization detector (FID). The system utilized a split–splitless injection method. Helium served as the carrier gas, flowing at a rate of 1 mL/min. The temperature program began at 100 °C, increased at a rate of 5 °C/min to 160 °C, followed by a ramp at 20 °C/min to 320 °C, and concluded with a 20-min hold. To quantify the chemical species present, the internal standard method was employed. Detailed descriptions of other operational conditions for the analysis are available in prior publications [36].

The analysis and monitoring of biodiesel samples were conducted using a titrator 702 SM Basic Titrino-Metrohm Ltd CH 9101. Herisau, Switzerland and in accordance with specific American Oil Chemistry Society (AOCS) standards: acid value (AV) following AOCS Ca 5a-40, moisture content using the Karl Fischer method, and viscosity (ν) as per ISO 3104. The oxidation stability of the ethyl esters was assessed using the Rancimat method, ASTM D97, with a 743 Rancimat-Metrohm AG CH-9100 (Herisau, Switzerland). Additionally, the cloud point (CP(°C)) and pour point (PP) of the ethyl esters were determined using an Automatic analyzer CFPP (Cloud and Pour point measurements ISL CPP 97-2), in line with the ASTM D2500 method.

Cold filter plugging point (CFPP) of ethyl esters were calculated by inserting measured CP(°C) values into the following equation: CFPP(°C)= CP-4,5(°C), developed by Dunn and Moser [37] and used for individual fatty acids methyl esters with a moderate standard error.

### 2.5 Ethyl esters synthesis

A big batch of ethyl esters was produced to carry on the different cold properties testing. The production of ethyl esters was carried out by the transesterification of waste cooking oil using enzymatic catalyst in the present of ethanol. The optimal conditions were based on a preliminary works that has been published elsewhere [17,33]. The optimal yields were obtained with a catalyst concentration of 4.2 %, an alcohol/oil molar ratio of 6:1, and an operating temperature of 36 °C. Under these conditions, the fatty acid ethyl ester (FAEE) yield exceeded 98 %.

**Fig. 1:**
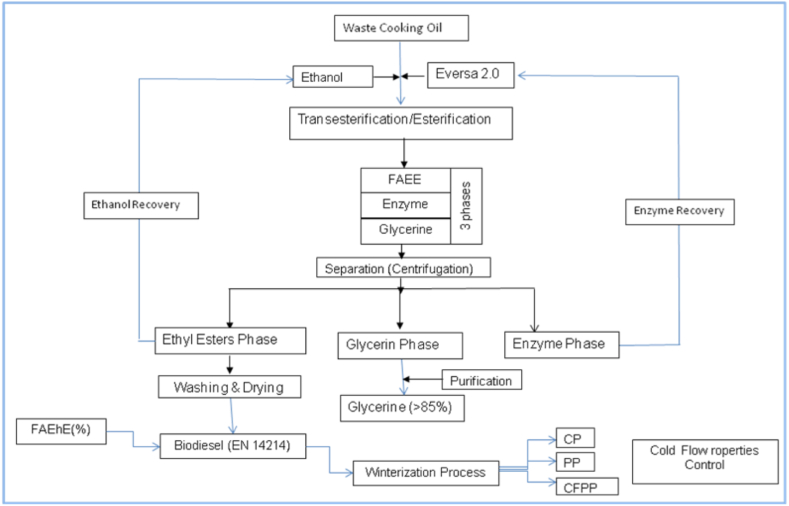
A schematic representation of the main processing steps for biodiesel production from high free fatty acids waste cooking oil.

Fig. 1 illustrates the primary steps involved in producing biodiesel from waste cooking oil high in free fatty acids. After the completion of the reaction time, the samples were decanted and separated to eliminate any glycerol and catalyst residues. The surplus alcohol was then removed through evaporation under reduced pressure for reuse. The ethyl ester layer underwent gentle water washing to cleanse any remaining glycerol and enzyme. The final product, WCO ethyl esters, had a water content of less than 0.02 %. The enzyme was separated from the glycerol layer using centrifugation.

### 2.6 Statistical analysis and improvement study of cold flow properties

#### 2.6.1 Statistical analysis

The enhancement of the cold flow properties of ethyl esters derived from waste cooking oil using an enzymatic catalyst was explored and refined through a factorial experimental design. This study utilized a comprehensive two-level factorial design, 2^3^, assessing three variables at two levels each, and expanded these findings using Response Surface Methodology (RSM). Implementing this methodology necessitated careful selection of the response variables, factors, and their levels.

The chosen response variables used in the RSM were the cloud point (CP), pour point (PP), and cold filter plugging point (CFPP) of the ethyl ester due to their relance for cold flow properties and the use of biodiesel in cold weathers. The variables that were varied to optimize the abovementioned parameters were cooling temperature (X_T_), cooling duration (X_t_), and the blending percentage of 2-ethylhexyl esters of fatty acids (FAEhE, X_FAEhE%_). The impeller's speed was maintained at 100 rpm and the reaction pressure was kept constant.

The determination of the levels for these factors was informed by preliminary studies and existing literature [38].

The highest cooling temperature level was set at 5 °C and the minimum cooling temperature level was fixed at −15 °C, this value was remaining constant at the selected value for each experiment. The cooling time varied from 15 to 45 min, which is sufficient to ensure that the phase transition was in the steady state, this time was measure after the cooling temperature has been reached. For blending, different levels of FAEhE were chosen, the levels varied from 5 wt% as low level and 15 wt% as high level of the whole mass sample based on literature data [38]. The blending was carried out after the winterizations process has been done in order to compare the effect of winterizations alone as well as winterizations combine with blending.

Table 2 displays the experimental matrix used in the factorial design. The initial three columns illustrate the coded factor levels as "±1″ in dimensionless coordinates, while the subsequent three columns present the factor levels in their natural units. All experimental trials were conducted in a random sequence. To estimate experimental error, four experiments were performed at the central point, which is coded as '0'.

**Table 2:** 2^2^ Factorial experiment matrix: Experimental results.

Experiment	Coded design levels			Real values			CP(°C)	PP(°C)	CFPP(°C)
	X_T_	X_t_	X_FAEhE%_	T(°C)	t(min)	FAEhE(%)			
1	−1	−1	−1	−15	15	5	−4.5	−7.0	−7.45
2	+1	−1	−1	5	15	5	−5.1	−6.2	−8.05
3	−1	+1	−1	−15	45	5	−5.0	−7.3	−7.95
4	+1	+1	−1	5	45	5	−4.4	−6.4	−7.34
5	−1	−1	+1	−15	15	15	−6.5	−8.4	−9.5
6	+1	−1	+1	5	15	15	−5.5	−8.0	−8.46
7	−1	+1	+1	−15	45	15	−6.6	−8.8	−9.6
8	+1	+1	+1	5	45	15	−6.0	−8.3	−8.96
9	0	0	0	−5	30	10	−6.3	−7.7	−10.68
10	0	0	0	−5	30	10	−5.9	−7.8	−10.78
11	0	0	0	−5	30	10	−6.1	−7.4	−9.67
12	0	0	0	−5	30	10	−6.2	−7.6	−10.58

## 3 Results and discussion

In this study, the production of biodiesel from waste cooking oil was carried out using ethanol and Eversa. Once the biodiesel was produced, winterizations and blending were tested as methods to improve the cold flow properties. As a first step, a factorial design was carried out to study the effect of three main variables, temperature (*X*_*T*_), cooling time (*X*_*t*_), and percentage of FAEhE added, (*X*_*FAEhE(%)*_) over the CP, PP, CFPP. The process was studied as a linear and non linear stage due to the statistical significant of the curvature of the data.

### 3.1 Linear stage

A 2^3^ factorial design was employed, augmented by four central points to assess experimental error. The outcomes are presented in Table 2. Statistical analysis was conducted on these experimental data, leading to the calculation of statistically significant effects and interactions among the three variables. The tests for statistical significance are shown in Table 3 Temperature (*X*_*T*_), cooling time (*X*_*t*_), and percentage of FAEhE added, (*X*_*FAEhE(%)*_) effects and their interactions were fitted by multiple regression analysis to a linear model. The response function for the main significant effects and interactions can be expressed by Eqs. (1), (2), (3):

(1) **CP** = −5,45 + 0,2* *X*_*T*_ - 0,05* *X*_*t*_ - 0,7* *X*_*FAEhE(%)*_ + 0,1* *X*_*T*_ * *X*_*t*_ + 0,2* *X*_*T*_ * *X*_*FAEhE(%)*_ - 0,1* *X*_*t*_ * *X*_*FAEhE(%)*_ r^2^ = 0.9208

(2) **PP** = −7,55 + 0,325* *X*_*T*_ - 0,15* *X*_*t*_ - 0,825* *X*_*FAEhE(%)*_ + 0,025* *X*_*T*_ * *X*_*t*_ - 0,1* *X*_*T*_ * *X*_*FAEhE(%)*_ - 0,025* *X*_*t*_ * *X*_*FAEhE(%)*_ r^2^ = 0.987

(3) **CFPP** = −8,41 + 0,2113* *X*_*T*_ - 0,0487* *X*_*t*_ - 0,7162* *X*_*FAEhE(%)*_ + 0,1012* *X*_*T*_ * *X*_*t*_ - 0,2087* *X*_*T*_ * *X*_*FAEhE(%)*_ - 0,1013* *X*_*t*_ * *X*_*FAEhE(%)*_ r^2^ = 0.818

**Table 3:** 2^2^ Factorial design for linear model: statistical analyses for cloud point (CP), pour point (PP)and cold filter plugging point (CFPP).

Responses	
**CP (°C)**	
**Main effects and interactions:**	
*X*_*T*_ = 0.4, *X*_*t*_ = −0.1, *X*_*%FAEhE*_ = −1.4	
*Signifiance of curvature*	
Curvature: *C = CP-CP*_C_*=* 0.53	
Confidence Curvature interval: ±0.25	Significance: Yes
Response equation	
CP = −5.45 + 0,2* X_T_ - 0,05* X_t_ - 0,7* X_FAEhE(%)_ + 0,1* X_T_ * X_t_ + 0,2* X_T_ * X_FAEhE(%)_ −0,1* X_t_ * X_FAEhE(%)_ r^2^ = 0.9208	
**PP (°C)**	
Main effects and interactions:	
*X*_*T*_ = 0.74, *X*_*t*_ = −0.2, *X*_*%FAEhE*_ = −1.74	
*Signifiance of curvature*	
Curvature: *C = PP-PP*_C_*=* 0.125	
Confidence Curvature interval: ±0.25	Significance: Slight effect
Response equation	
PP = −7.55 + 0.325 *X*_T_ - 0.15*X*_t_ - 0.825 *X*_%FAEhE_ + 0.025*X*_Tt_ - 0.10 *X*_T*%FAEhE_ −0.025 *X*_t*%FAEhE_ r^2^ = 0.987	
**CFPP (°C)**	
Main effects and interactions:	
X_T_ = 0.2, X_t_ = 0.36, X_%FAEhE_ = −1.2	
*Signifiance of curvature*	
Curvature: *C = CFPP-CFPP*_C_*=* 2.1	
Confidence Curvature interval: ±0.73	Significance: Yes
Response equation	
CFPP = −8,41 + 0,2113 *X*_T_ - 0.0487 *X*_t_ - 0.7162 *X*_FAEhE(%)_ + 0.1012*X*_T*t_ + 0.2087 *X*_T*FAEhE(%)_ - 0.1013 *X*_t*FAEhE(%)_ r^2^ = 0.818	

The most significant factor is the percentage of FAEhE added (*X*_*FAEhE(%)*_) with a negative effect on the CP, PP and CFPP responses. In addition, there is a significant curvature effect for CP, CFPP and slightly significant curvature effect for PP. Therefore, it was necessary to consider the data for the nonlinear stage and fit the data to a second-order model.

### 3.2 Non-linear stage

Due to the significant curvature effect observed in the linear phase, a second-order model was necessary for CP, PP, and CFPP. To accommodate this, star points representing additional experimental points were integrated into the two-level factorial design for the three critical factors: cooling temperature (XT), cooling time (Xt), and the percentage of FAAE added (XFAEhE%). The comprehensive central composite design, derived from Box and Hunter [39], incorporates factorial points, center points, and star points, as detailed in Table 4. The corresponding model is the complete quadratic surface between the response and the factors.

**Table 4:** Experimental results of the star points.

Experiment	Coded design levels			Real Values			CP(%)	PP(°C)	CFPP(°C)
	X_T_	X_t_	X_FAEhE%_	T(°C)	t(min)	FAEhE(%)			
13	+α	0	0	11.8	30	10	−7.4	−12.5	−10.37
14	-α	0	0	−21.8	30	10	−8.2	−15.2	−13.00
15	0	+α	0	−5	55.2	10	−7.0	−12.2	−9.97
16	0	-α	0	−5	4.8	10	−9	−12	−11.99
17	0	0	+α	−5	30	18.4	−10	−15	−13.00
18	0	0	-α	−5	30	1.6	−8	−13	−10.98

To create a central composite design, six extra experiments, known as star points and encoded as ±α, were incorporated into the 2^3^ factorial design along with center points. Here, α represents the distance from the origin to a star point, calculated as α = 2^(n/4)^, which in this case equals 1.68. The coefficients of Equation (4) were identified through multiple regression analysis, considering all independent variables and their interactions, irrespective of their significance levels. The most accurate response surfaces were represented using the following statistical model:

(4) **CP(°C)** = −7,51 + 0,2157*X*_*T*_ +0,2170*X*_*t*_ - 0,6563*X*_*FAEhE(%)*_ +0,39*X*_T_^2^ + 0,1*X*_*T*_*X*_*t*_ + 0,2 *X*_*T*_*X*_*FAEhE(%)*_ + 0,3193 *X*_*t*_^*2*^ - 0,1 *X*_*t*_*X*_*FAEhE(%)*_ - 0,0343 *X*_*FAEhE(%)*_^*2*^ r^2^ = 0.852

(5) **PP(°C)** = −10.51 + 0,5229*X*_*T*_ - 0,1125*X*_*t*_ - 0,7296*X*_*FAEhE(%)*_ - 0,1636*X*_T_^2^ + 0,025*X*_*T*_*X*_*t*_ - 0,1 *X*_*T*_*X*_*FAEhE(%)*_ + 0,4552 *X*_*t*_^*2*^ - 0,025 *X*_*t*_*X*_*FAEhE(%)*_ - 0,2166 *X*_*FAEhE(%)*_^*2*^ r^2^ = 0.937

(6) **CFPP(°C)** = −11.94 + 0,4476*X*_*T*_ + 0,2202*X*_*t*_ - 0,6683 *X*_*FAEhE(%)*_ + 0,6241*X*_T_^2^ + 0,1012*X*_*T*_*X*_*t*_ + 0,2087 *X*_*T*_*X*_*FAEhE(%)*_ + 0,8734*X*_*t*_^*2*^ - 0,1013*X*_*t*_*X*_*FAEhE(%)*_ + 0,5163*X*_*FAEhE(%)*_^*2*^ r^2^ = 0.857

### 3.3 Cloud point (CP)

#### 3.3.1 Influence of temperature

**Fig. 2:**
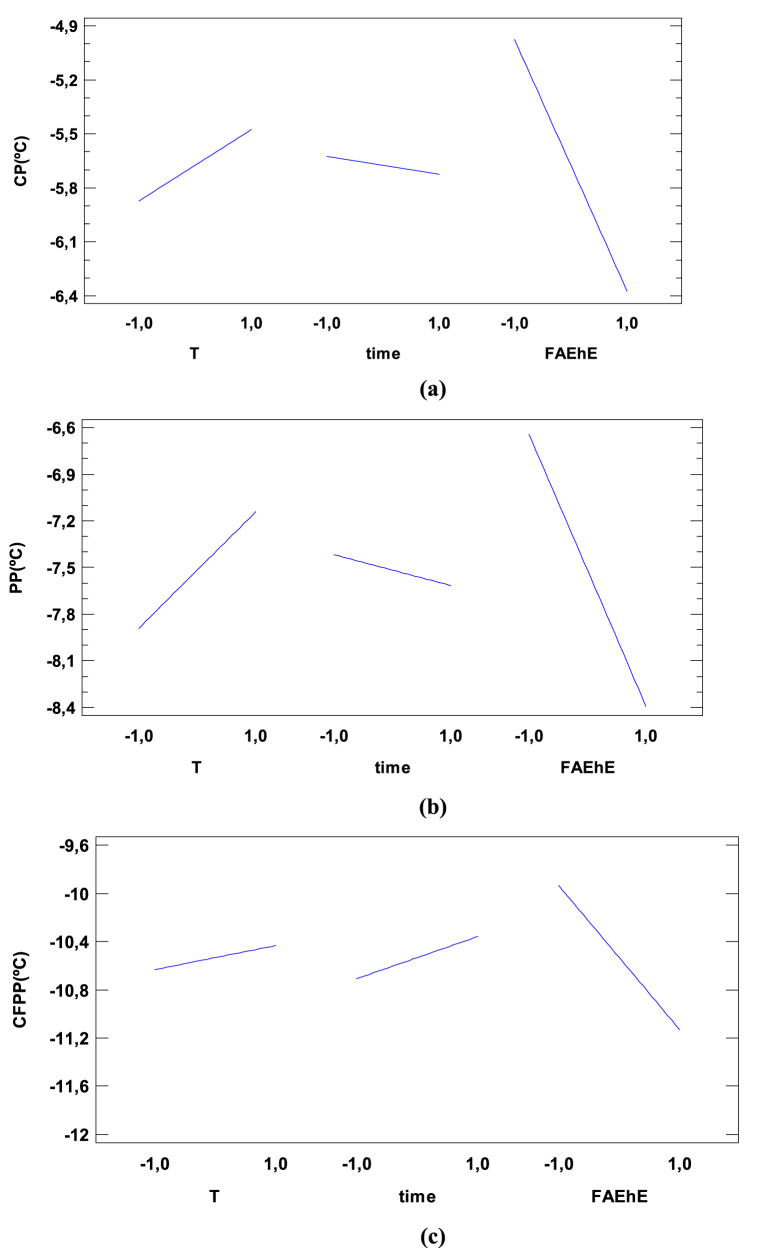
Main Effect for CP, PP and CFPP response.

In both models linear as well as the quadratic, the influence of cooling temperature is statistically significant in the studied range (−15 – 5 °C). This effect has a positive influence in the CP response as it can be seen in Fig. 2a.

#### 3.3.2 Cooling time

The influence of the cooling time in CP is evaluated by increasing the time from 15 to 45 min. The analysis shows that the cooling time is a significant factor affecting CP with a slight negative influence on the response as it is presented in Fig. 2a.

#### 3.3.3 Percentage of FAEhE added

The percentage of FAEhE added is the most influent variable in the CP response. This influence has a negative effect in the CP response. The increase in the percentage of FAEhE is causing a change in the response decreasing the CP, from −5 to −6.5 °C as presented in Fig. 2a.

### 3.4 Pour point (PP)

#### 3.4.1 Influence of temperature

The influence of cooling temperature on PP is statistically significant in the range (−15– 5 °C). This effect has a positive influence in the PP as it can be seen in Fig. 2b where an increasing the cooling temperature produce and increase of PP from −8.0 to −7.1 °C.

#### 3.4.2 Cooling time

The influence of the cooling time on PP is evaluated by increasing the time from 15 to 45min. The cooling time is a slightly significant factor affecting PP with a negative influence. A decrease of PP from −7,4 to −7,8 °C was observed and presented in Fig. 2b.

#### 3.4.3 Percentage of FAEhE added

The influence of percentage of FAEhE added on PP response is evaluated by increasing the percentage of FAEhE from (5–15 %) which turned out to be the most influent variable in this case as it was in the previous analysis. This translates into an increase in this factor decreases the PP from −6,7 to −8,4 °C as shown in Fig. 2b.

### 3.5 Cold filter plugging point (CFPP)

#### 3.5.1 Influence of temperature

The influence of cooling temperature on cold filter plugging point (CFPP) is statistically significant in the studied range (−15– 5 °C). This effect has a slight positive influence in the CFPP. As can be seen in Fig. 2(c) an increase in cooling temperature from −15 to 5 °C increases slightly the CFPP from −10.8 to −10.4 °C.

#### 3.5.2 Cooling time

The influence of the cooling time on CFPP was evaluated by increasing the time from 15 to 45 min. The cooling time is a moderate significant factor affecting CFPP with a positive influence on the response. An increase of CFPP from −10,8 to −10,4 °C was observed.

#### 3.5.3 Percentage of FAEhE added

The statistical analysis shows that within the experimental range, the addition of FAEhE is the most significant factor affecting CFPP value with a negative effect. The influence of percentage of FAEhE added on CFPP response is evaluated by increasing its value from 5 to 15 %, this resulted in a variation of the CFPP from −10,0 to −11,2 °C as it was presented in Fig. 2c.

### 3.6 Analysis of responses: CP, PP and CFPP

**Fig. 3:**
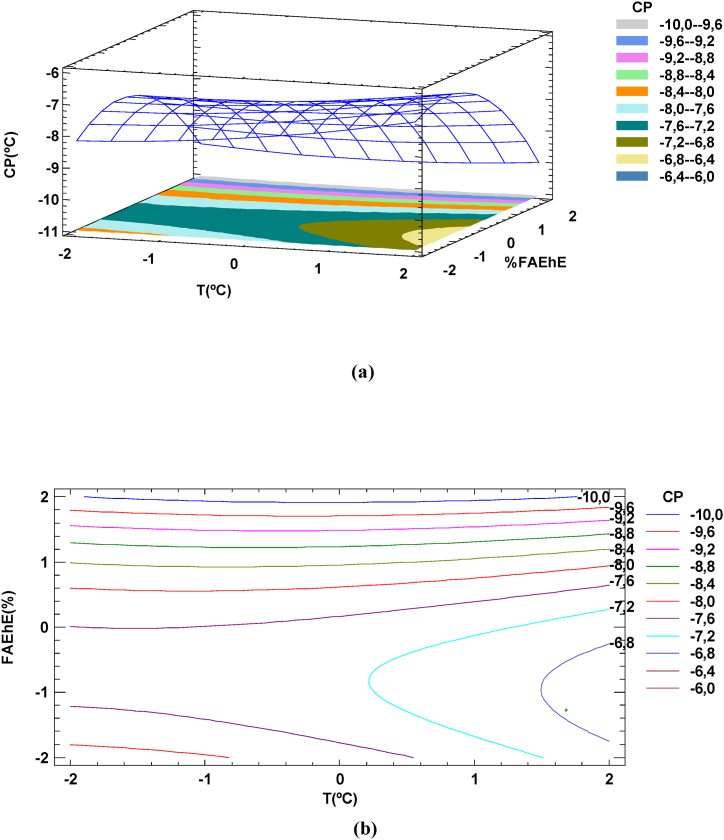
Response surface and contour plot of ester yield us cooling temperature and % of FAEhE added surface (a) and contour plot (b) for CP. cooling time, t = 30 min.

The significance of the statistical model can be visualized by representing the responses (CP, PP and CFPP) as a function of the three important factors cooling temperature, *X*_*T*_, cooling time, *X*_*t*_ and percentage of FAEhE added, *X*_*FAEhE(%)*_. The surface and the contour plot of CP versus cooling temperature, *X*_*T*_ and percentage of FAEhE added, *X*_*FAEhE*_ obtained when individual experimental data was used is shown in Fig. 3(a) and Fig. 3(b). The contour plot shows that working with a cooling temperature of -5 °C, adding a 10 % of FAEhE and cooling during 30 min a cloud point of (*CP* = −8 °C) could be achieved.

**Fig. 4:**
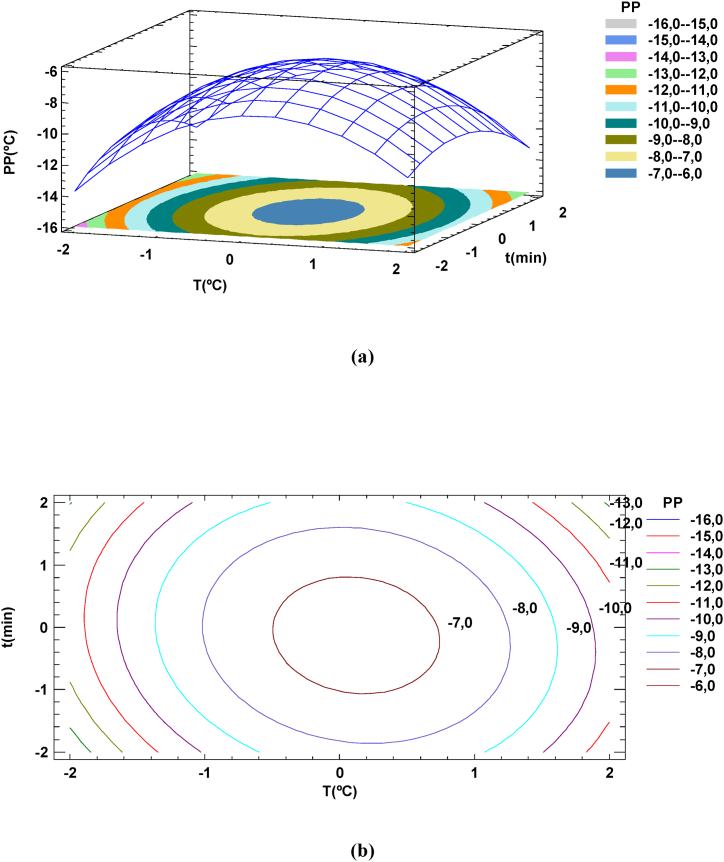
Response surface and contour plot of ester yield us cooling temperature and cooling time surface (a) and contour plot (b) for PP. % of FAEhE added = 10 %.

Respect to the pour point, the surface, and the contour plot of PP versus cooling temperature, *X*_*T*_ and cooling time, *X*_*t*_ obtained when individual experimental data was used is shown in Fig. 4(a) and Fig. 4(b). The contour plot shows that the lowest pour point (PP = −15 °C) could be achieved, working with a low level of cooling temperature (−15 °C), adding a 15 % of FAHE and cooling during 30 min.

**Fig. 5:**
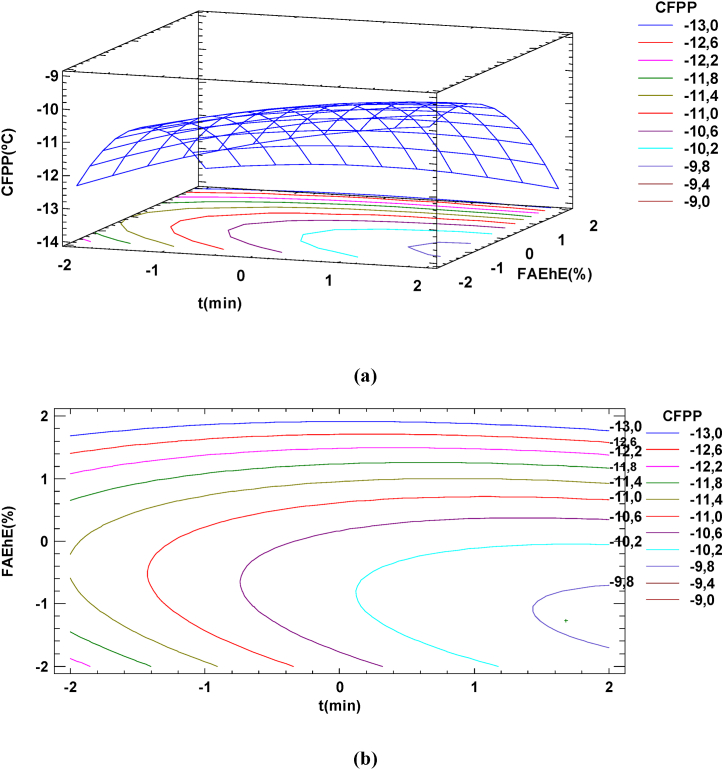
Response surface and contour plot of ester yield us cooling time and % of FAEhE added, surface (a) and contour plot (b) for CFPP. cooling temperature, T = −5 °C.

Regarding the Cold filter plugging point, the surface and the contour plot of CFPP versus cooling time, *X*_*t*_ and percentage of FAEhE added, *X*_*FAEhE(%)*_ obtained when individual experimental data was used is shown in Fig. 5(a) and Fig. 5(b). The contour plot shows that the lowest Cold filter plugging point (CFPP = −12 °C) could be achieved, adding a 15 % of FAEhE and cooling at -5 °C during 20 min.

However, from an economical point of view, working with a cooling temperature of (-5 °C), adding a 10 % of FAEhE and cooling during 30 min should be elected. According to these conditions, CP of −7.5 °C, CFPP of −10 °C and a PP of −12 °C could be obtained.

The results obtained in this study also show an average reduction of 20,63 wt% and 8,64 wt% of saturated palmitic (C16:0) and stearic (C18:0) fatty acids ethyl esters (FAEE) respectively. The loss of palmitic acid is higher as compared to stearic acid; the possible reason is that the melting point of palmitic is lower than that of stearic acid. Also, the solubility of palmitic acid is lesser as compared to stearic acid.

**Fig. 6:**
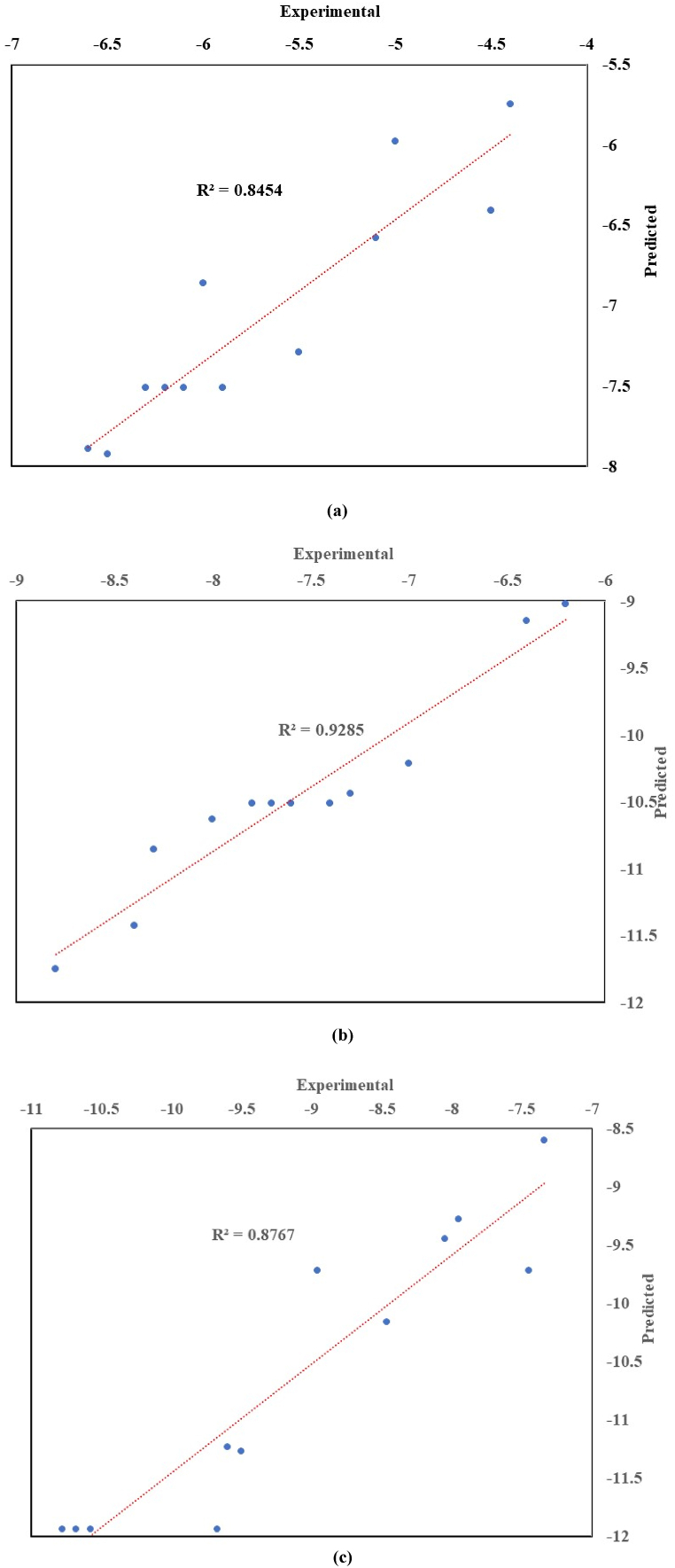
Plots of experimental vs predicted for the second order model for the responses. (a): CP, (b): PP and (c): CFPP.

Fig. 6 (a, b, & c) displays the comparison of experimental and predicted values for the response variables CP, PP, and CFPP. The fitting is quite good for all systems considered and all variables studied as it can be seen from the R^2^ values in the plot.

### 3.7 Quality control of biodiesel

Viscosity, acid value, ester contents, oxidation stability, cloud point, pour point and cold filter plugging point are shown in Table 5. In this table it is presented the value of the biodiesel from waste cooking oil without any treatment as well as the best biodiesel produced from waste cooking oil follow by winterizations and optimizations based on the DOE and RSM performed, given the best cooling temperature and blending ratio and in line with optimal conditions published elsewhere [17,33]. In addition, a column with then 14214 values is also presented. Most of the experimentally determined values agreed with this European Biodiesel Standard Norm EN14214.

**Table 5:** Quality control of WCO fatty acids ethyl ester before after treatment compared to EN 14214.

Properties	WCOB Before	WCOB After	EU Standard, EN 14214
Viscosity at 40 °C	5.3	6,7	Max. 5.00 mm^2^/s
Acid value (mg KOH/g)	0.37	0.45	Max. 0.50 mg KOH/gr
Iodine value	115.2	116	120
Water content mg/kg	200	50	Max. 500 mg/kg
Ester contents (wt.%)	98.54	96.7	Min. 96.5 % (m/m)
Monoglyceride content (wt.%)	0.75	0.1	Max. 0.80 % (m/m)
Diglyceride content (wt.%)	0.15	0.2	Max. 0.20 % (m/m)
Triglyceride content (wt.%)	0.02	0.05	Max. 0.20 % (m/m)
Free glycerol (wt.%)	0.013	0.01	Max. 0.02 % (m/m)
Oxidation Stability (h)	7.3	4.5	>8h
Cloud point (°C)	−3.00	−9	-a
Pour point (°C)	−7.00	−15	-a
Cloud filter plugging point (°C)	−5.00	−12	Depending on the country

a Not specified. EN 14214 uses time and location dependent values for the cold filter plugging point (CFPP) instead.

The acid value recorded was 0.45 mg KOH/g, well below the normative maximum of 0.5 mg KOH/g, and the kinematic viscosity measured 6.7 mm^2^/s at 40 °C, closely aligning with the specified range. The slight increase in viscosity may be attributed to the addition of % FAEhE branched esters. This characteristic could be enhanced by mixing the biodiesel with diesel in ratios of approximately 5–10 %.

The operational performance of biodiesel at low temperatures is typically assessed using three key parameters: cloud point (CP), pour point (PP), and cold filter plugging point (CFPP). Among these, CFPP is often regarded as the most dependable measure of low-temperature operability. This is because, once the CFPP is reached, the fuel begins to form solids large enough to clog the fuel filter, potentially leading to engine failure [40].

**Fig. 7:**
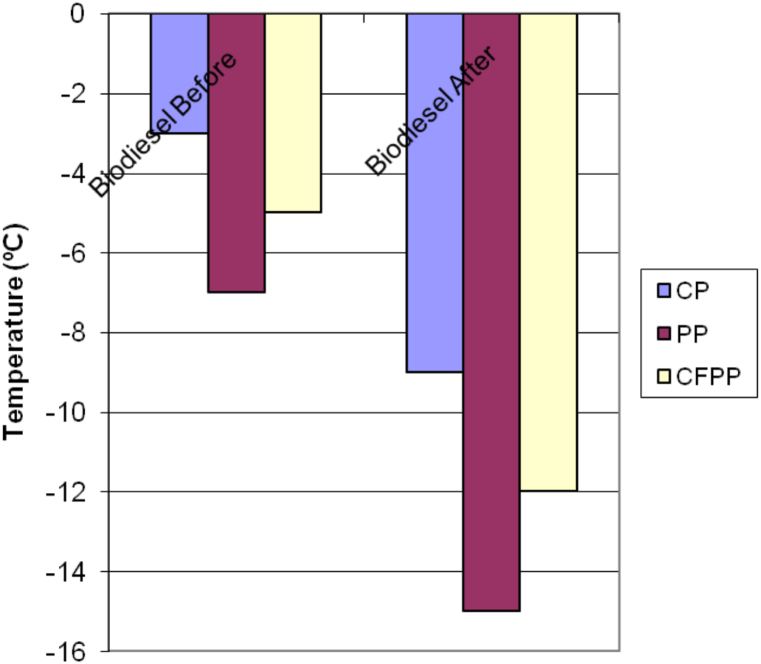
The CP, PP and CFPP of biodiesel samples before and after treatment of WCOB.

Fig. 7 illustrates the cloud point (CP), pour point (PP), and cold filter plugging point (CFPP) of biodiesel samples before and after undergoing treatment. Post-treatment, the waste cooking oil (WCO) biodiesel samples exhibited improved cold flow properties, with a CP of −9 °C, a PP of −15 °C, and a CFPP of −12 °C. These results demonstrate the effectiveness of using a dual approach—combining controlled winterization and the addition of synthetically produced branched fatty acid alkyl esters (FAEhE)—in enhancing the cold flow characteristics of biodiesel derived from high free fatty acid waste cooking oil, as reflected in the CP, PP, and CFPP values compared to those prior to the enhancement process.

The oxidative stability of WCO biodiesel was evaluated using the Rancimat method as per EN14214, with the average result from two tests being approximately 4.5 h. This indicates that the biodiesel samples possess limited resistance to oxidative degradation by free radicals, exhibiting Induction Period (IP) values between 4 and 5 h. In terms of the Rancimat test, biodiesel derived from waste cooking oil showed inadequate oxidative stability. Furthermore, the improved biodiesel samples failed to fulfill the oxidative stability criteria set by the EN14214 standard. The IP values of the samples were influenced by their fatty acid compositions. The introduction of commercial, either synthetic or natural, antioxidants could enhance the oxidation stability of the tested biodiesel fuel [41].

Regarding the EN 14214 standard, the monoglyceride (MG) content in biodiesel should not exceed 0.8 wt%, and the levels of diglycerides (DG) and triglycerides (TG) should each be below 0.2 wt%, while the ester content must be at least 96.5 wt%. The ester content of the WCO biodiesel was found to be 96.7 %, with the concentrations of individual glycerides (MG, DG, TG) meeting these requirements. Following the cold flow enhancement process, the treated biodiesel is suitable for direct use across both cold and warm climates. The cold flow characteristics of the biodiesel, including the cloud point (CP), pour point (PP), and cold filter plugging point (CFPP), were notably improved by the method developed in this study, which involves cooling the sample to.

-5 °C for 30 min and incorporating 10 % of FAEhE.

## 4 Conclusions

In the present waste cooking oil was transformed into biodiesel using ethanol and Eversa as catalysts. The cold flow properties of the biodiesel produced were studied. The variables studied were cloud point (CP), pour point (PP) and could filter plugging point (CFPP).

Improvements to these properties were achieved after winterizations and after an optimization study, for this, a RSM was carried out and the effects of cooling temperature, *X*_*T*_, cooling time, *X*_*t*_ and percentage of fatty acids 2-ethylhexyl esters (FAEhE) added, *X*_*FAEhE(%)*_ on CP, PP and CFPP of biodiesel obtained for the optimized system. The final biofuel fulfill most specifications of European Union Standards (EN14214).

It has been found that applying winterization only to the biodiesel produced by transesterification of waste cooking oil using ethanol as the alcohol can enhance its operability in cold weather. This approach reduces the cloud point (CP) by 6 °C, the pour point (PP) by 8 °C, and the cold filter plugging point (CFPP) by 7 °C.

Further studies to improve fuels properties as well as to evaluate emissions and performance could be based on the outcome of this manuscript.

## Data availability

Data will be made available on request.

## CRediT authorship contribution statement

**Abderrahim Bouaid:** Writing – original draft, Validation, Supervision, Resources, Methodology, Investigation, Conceptualization. **Gabriel Iliuta:** Visualization, Validation, Resources, Formal analysis, Data curation. **Jorge Mario Marchetti:** Writing – review & editing, Writing – original draft, Visualization, Project administration, Funding acquisition.

## Declaration of competing interest

The authors declare that they have no known competing financial interests or personal relationships that could have appeared to influence the work reported in this paper.

## Nomenclature

CP: cloud point
PP: pour point
CFPP: cold filter plugging point
FAEE: fatty acid ethyl esters
WCOB: waste cooking oil biodiesel
t: cooling time
T: cooling temperature, °C
X_i_: level of factor i
Y: ester conversion, %
n: number of factors in a factorial design
r: correlation coefficient
t: Student's t value
s: standard deviation
α: distance from origin to star point in a central composite design
Y: mean response for the factorial design
Y_c_: mean centrepoints response
a_0_: intercept
a_k_: first-order model coefficient
a_kk_: quadratic coefficient for the ith variable
a_kj_: interaction coefficients for the interaction of variables k and j
X_k_: independent variables
